# Comment on Balsamo et al.: Birt-Hogg-Dubé syndrome with simultaneous hyperplastic polyposis of the gastrointestinal tract: case report and review of the literature

**DOI:** 10.1186/s12920-022-01229-5

**Published:** 2022-04-15

**Authors:** Irma van de Beek, Maurice A. M. van Steensel, Arjan C. Houweling

**Affiliations:** 1 Department of Human Genetics, Amsterdam UMC, Vrije Universiteit Amsterdam, De Boelelaan 1117, Amsterdam, The Netherlands; 2 Lee Kong Chian School of Medicine, Nanyang Technological University, 50 Nanyang Avenue/Singapore Skin Research Institute of Singapore, Agency for Science, Technology and Research, 11 Mandalay Road, Singapore, Singapore

**Keywords:** Birt-Hogg-Dubé syndrome, Hyperplastic polyposis

## Abstract

The publication by Balsamo and colleagues describes a patient with Birt-Hogg-Dubé syndrome and hyperplastic polyposis throughout the gastro-intestinal tract. We question whether the diagnosis of BHD in this patient was justified. Using the previously proposed diagnostic criteria for establishing the diagnosis of BHD as a guideline, we systematically describe our concerns. In our opinion, the patient described by Balsamo and colleagues does not meet any of the proposed major and minor criteria for the diagnosis of Birt-Hogg-Dubé syndrome. Therefore, we believe that it is not justified to suggest a possible association between hyperplastic polyposis and Birt-Hogg-Dubé syndrome based on this patient, even though a higher risk for colorectal polyposis in Birt-Hogg-Dubé syndrome has not been excluded so far.

## Background

Birt-Hogg-Dubé syndrome (BHD, MIM# 135,150) is a rare disorder with autosomal dominant inheritance. It is caused by mutations in the *FLCN* gene [1, 2]. The skin phenotype, consisting of multiple fibrofolliculomas, was first described in the 1970s [3, 4]. Fibrofolliculomas consist of proliferation of specialized connective tissue surrounding an abnormal hair follicle, in which epithelial strands extend out into the connective tissue mantle [5–7]. Subsequently, many case reports described several other features that could be associated with BHD. However, it was not until 2002 that the association of BHD with pneumothorax, lung cysts and renal tumours was confirmed [8]. Specifically in rare genetic diseases, case reports on associated features are of high importance and might lead to the discovery of additional phenotypic characteristics of the disease which may allow preventive measures or surveillance. It was therefore with considerable interest that we read the publication by Balsamo and colleagues, in which a patient with BHD and hyperplastic polyposis throughout the gastro-intestinal tract was described [9]. We question whether the diagnosis of BHD in this patient was correct given the absence of specific clinical features of BHD and no (likely) pathogenic *FLCN* mutation. Therefore the suggested association between BHD and hyperplastic polyposis might not be justified. Using the previously proposed diagnostic criteria for establishing the diagnosis of BHD as a guideline, we will further elaborate on this subject [10].

## FLCN pathogenic variants

The identification of a pathogenic variant in the *FLCN* gene is sufficient to establish the diagnosis of BHD. *FLCN* sequencing was performed in the patient described by Balsamo and colleagues but revealed no (likely) pathogenic mutation. As far as we can discern, no analysis for large intragenic deletions or duplications (such as MLPA) was performed, even though these have been reported in BHD patients [11, 12]. An intronic variant was detected (c.1538 + 14 T > G), but this variant was classified as likely benign by the authors and therefore cannot be of use in the diagnosis of BHD in this patient. The variant is present in 2.4% of African healthy control alleles and can therefore likely be classified as non-pathogenic [13]. However, the lack of a mutation in *FLCN* does not completely exclude BHD. Pathogenic variants in *FLCN* are detected in up to 95% of patients with a clinical diagnosis of BHD based on the literature and our own experience [12, 14, 15]. The remaining patients might represent cases of BHD due to a currently unidentifiable variant in *FLCN*, for example a deep intronic one or a variant in a regulatory sequence of *FLCN*. In some families previously described as having BHD, another diagnosis can now be established, for example familial multiple discoid fibromas [16].

## Fibrofolliculomas

The presence of fibrofolliculomas is considered pathognomonic for BHD. One of the proposed criteria for diagnosing BHD is the presence of at least five fibrofolliculomas or trichodiscomas, of which at least one is histologically confirmed [10]. Of note, fibrofolliculomas and trichodisomas in BHD are considered to be different histologic aspects of the same lesion as seen at different section levels, while trichodiscomas without the typical histological features of fibrofolliculomas have been described in individuals with familial multiple discoid fibromas [6, 16]. Balsamo and colleagues describe that their patient had skin lesions which were considered trichodiscomas at anatomopathological examination. However, the histological image in Fig. 1 in the paper is showing a fibroepithelial polyp without the typical characteristics of a trichodiscoma or fibrofolliculoma. Fibroepithelial polyps (also called skin tags or acrochordons) are very common in the general population [17]. If this image is representative for all the skin lesions of the patient, his skin lesions are, in our opinion, not consistent with the diagnosis of BHD. If the patient did actually have multiple trichodiscomas which are not shown in the original paper, the diagnosis of BHD should be considered, as well as the alternative diagnosis of familial multiple discoid fibromas.

## Minor criteria

The proposed diagnostic criteria describe minor criteria of which two are required for the clinical BHD diagnosis: (1) multiple lung cysts, (2) renal cancer with early onset, multifocal, bilateral or specific histology and (3) a first-degree relative with BHD [10]. The patient described by Balsamo and colleagues has no family history of BHD and no lung cysts. He has two renal masses showing features suggestive of angiomyolipoma (AML) on MRI. Renal AMLs are not uncommon in the general population and most occur sporadically. For example, they have been detected in 0.13% of healthy adults and 2.2% of potential kidney donors [18, 19]. Renal AMLs are much more common in patients with tuberous sclerosis complex, which is caused by germline mutations in *TSC1* and *TSC2* [20, 21]. Several case reports have described AMLs in patients with BHD [22–25]. The involvement of both *TSC1/2* and *FLCN* in the mTOR pathway, might be a reason to suspect a causal connection between BHD and AMLs. This has, however, not been recognized in larger studies on renal tumours in BHD patients [26–29]. Furthermore, while there is a clear activation of mTOR upon loss of *TSC1* or *TSC2*, the effects of loss of FLCN on mTOR are dependent on the context and cell-type [30, 31]. In one of the case reports of a BHD patient with an AML, the authors show that the AML is likely sporadic, as a result of somatic loss of the *TSC* genes [25]. Altogether, the case reports more likely represent a coincidental co-occurrence of AML and BHD.

## Hyperplastic polyposis

Hyperplastic polyposis (more recently referred to as serrated polyposis syndrome (SPS)) presents with multiple and/or large serrated polyps throughout the colon. Although RNF43 germline mutations have been shown in a small proportion of (familial) cases of SPS [32–36], most cases of SPS occur sporadically and seem to have a multifactorial cause since both environmental and genetic risk factors have been shown to play a role [37, 38]. The association between BHD and colorectal polyps and/or carcinoma has been unclear for a long time. In a recent study, we did not find evidence for an increased risk for colorectal carcinoma in BHD. Furthermore, the ratio between adenomatous and hyperplastic polyps in BHD patients was comparable to that in individuals without BHD [39]. Other cohort studies also did not recognize hyperplastic polyposis as an important feature of BHD [8, 12, 27]. Therefore, an association between hyperplastic polyposis and BHD seems unlikely, but cannot be excluded.

## Other medical history

Although some of the other conditions in the patient described by Balsamo et al. have been reported in BHD, an association between these features and BHD has not been confirmed in larger groups and they are not useful in establishing BHD.

## Conclusion

In our opinion, Balsamo and colleagues have not provided information that is enough for establishing the diagnosis of BHD in their patient. In fact, none of the proposed clinical major and minor criteria are met. Therefore, we believe that it is not justified to suggest a possible association between hyperplastic polyposis and BHD based on the provided information about this patient. If the patient would meet the diagnostic criteria for BHD based on data not yet provided, the co-occurrence of BHD and hyperplastic polyposis might still be co-incidental but could give rise to further studies.

## Data Availability

Not applicable.

## Abbreviations

AML: Angiomyolipoma
BHD: Birt-Hogg-Dubé syndrome
SPS: Serrated polyposis syndrome
